# Dark side of anthocyanin pigmentation

**DOI:** 10.1111/plb.70047

**Published:** 2025-05-28

**Authors:** K. Wolff, B. Pucker

**Affiliations:** ^1^ Plant Biotechnology and Bioinformatics Institute of Plant Biology & BRICS, TU Braunschweig Braunschweig Germany; ^2^ Institute for Cellular and Molecular Botany University of Bonn Bonn Germany

**Keywords:** Anthocyanins, flavonoids, flower colour, pigmentation, secondary plant metabolites, specialized plant metabolites, stress response

## Abstract

Dark pigmentation can be observed in various parts of the plant, ranging from foliage to petals and berries. Here, we review the available knowledge about dark pigmentation in plants and the potential for biotechnological applications. The molecular basis of black pigmentation appears to vary among species, with anthocyanins playing a significant role, although specific anthocyanin types and their mechanisms differ. These findings suggest that the development of phenotypes is species‐specific or varies between larger taxonomic groups; this is further supported by the polyphyletic nature of dark pigmentation. Additionally, several different regulatory mechanisms have been described for the occurrence of dark pigmentation. First, the repression or knockout of the competing flavone biosynthesis has been shown to lead to darker pigmentation while another mechanism is based on the activation and upregulation of the anthocyanin biosynthesis genes in the presence of MYB transcription factors. Potential ecological functions of dark pigmentation were identified as protection of the photosynthesis apparatus, camouflage against herbivores, and the attraction of pollinators. Promising industrial applications include microbial factories for the production of natural food colourants, induction of novel phenotypes for the ornamental plant industry and, lastly, increase of anthocyanins within agriculturally relevant crops. Understanding the genetic basis of dark pigment accumulation would facilitate biotechnological and agricultural applications.

## Graphical abstract

This review examines dark anthocyanin pigmentation in plants by outlining its distribution, exploring the genetic mechanisms behind its development, and discussing its ecological role together with its potential for various industrial applications.

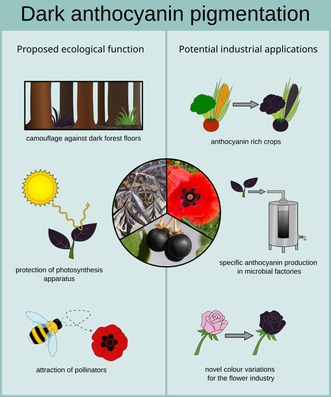

## INTRODUCTION

The process by which plants produce colour is a fascinating area of scientific inquiry that has captivated botanists, molecular biologists, and ecologists alike. The diverse range of colourful pigments found in plants serve vital functions, from photosynthesis facilitation to ecological interactions, and stress response. Flavonoids are one of the most studied specialized metabolites within plants and are responsible for the introduction of colour into various parts of the plant, such as petals, berries, or foliage (Winkel‐Shirley 2001). Anthocyanins form a diverse group of compounds derived from flavonoid biosynthesis. They can create a colour spectrum from red to blue, based on slight structural variations. The visual phenotype has led to the emergence of anthocyanin biosynthesis as one of the best studied specialized metabolite pathways in plants.

### Structure and biosynthesis of anthocyanins

**Fig. 1:**
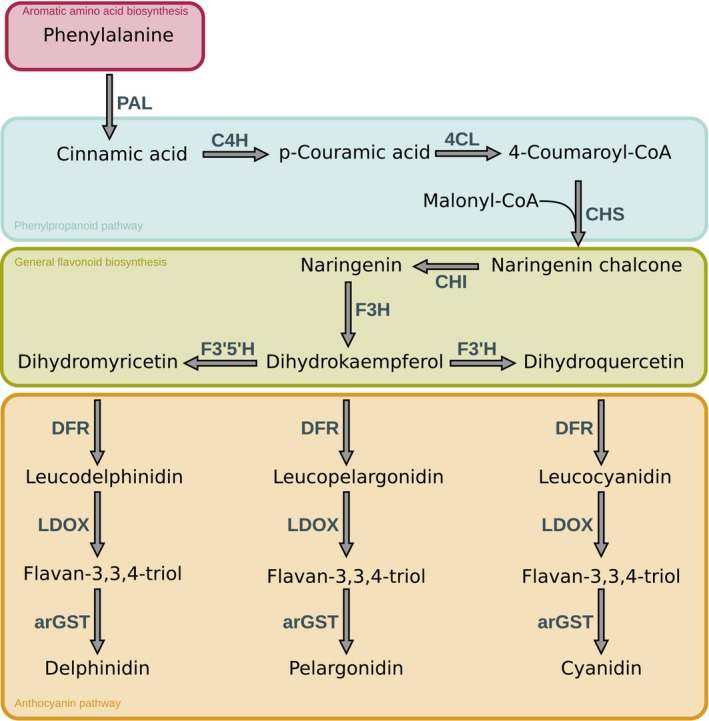
Simplified overview of the main reactions of the general anthocyanin pathway. The enzymes involved in synthesis are abbreviated: PAL, phenylalanine ammonia‐lyase; C4H, cinnamic acid 4‐hydroxylase; 4CL, 4‐coumarate‐CoA ligase; CHS, chalcone synthase; CHI, chalcone isomerase; F3H, flavanone 3‐hydroxylase; F3′H, flavonoid 3′‐hydroxylase; F3′5′H, flavonoid 3′,5′‐hydroxylase; DFR, dihydroflavonol 4‐reductase; LDOX, leucoanthocyanidin dioxygenase; arGST, anthocyanin‐related glutathione S‐transferase.

Anthocyanins have a basic structure consisting of C6‐C3‐C6 as the basic C skeleton structure. Six major anthocyanin groups have been reported in plants: delphinidin, pelargonidin, cyanidin, malvidin, petunidin, and peonidin (Houghton *et al*. 2021). The distinction is based on the number of hydroxyl groups, and their specific modifications through sugar or acetyl groups resulting in their respective colour properties (Winkel‐Shirley 2001). The biosynthetic pathway responsible for the formation of various anthocyanins is a well‐studied mechanism that starts with the amino acid phenylalanine. Anthocyanins are synthesized through a series of enzymatic catalytic reactions (Fig. 1). In the initial six steps, starting from phenylalanine, the enzymatic reactions involved in the construction of anthocyanins are consistent across the different types of anthocyanins. These reactions lead to the formation of dihydrokaempferol, which serves as a precursor for the subsequent steps. The pathway splits into three parallel branches to form the three anthocyanins cyanidin, pelargonidin, and delphinidin, which in turn are differentiated into malvidin, petunidin, and peonidin based on specialized reactions (Schwinn *et al*. 2014). Each subclass of the anthocyanins is able to produce a specific colour range, which is further influenced based on environmental conditions, especially the pH value, and additional modifications (Van Buren *et al*. 1974).

The general structure and decoration groups of the specialized anthocyanins are shown in Table 1. While the main structure of all six anthocyanins remains the same, they display varying residues at positions R_3_′ and R_5_′. These subtle differences are responsible for the ability of each anthocyanin to provide a certain colour.

**Table 1:** Overview of the most abundant anthocyanins and their chemical structure.

anthocyanin	*R*_3_′	*R*_5_′	chemical structure
Pelargonidin	−H	−H	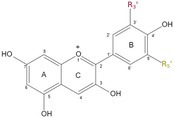
Cyanidin	−OH	−H	
Delphinidin	−OH	−OH	
Peonidin	−OCH_3_	−H	
Petunidin	−OH	−OCH_3_	
Malvidin	−OCH_3_	−OCH_3_	

This review article aims to provide an overview of the occurrence of dark pigmentation within the plant kingdom and to analyse current scientific knowledge of the underlying mechanisms as well as ecological functions. Further, it provides an outlook on future potential industrial applications and promising research directions.

### Dark pigmentation in plants – Where does it occur?

**Fig. 2:**
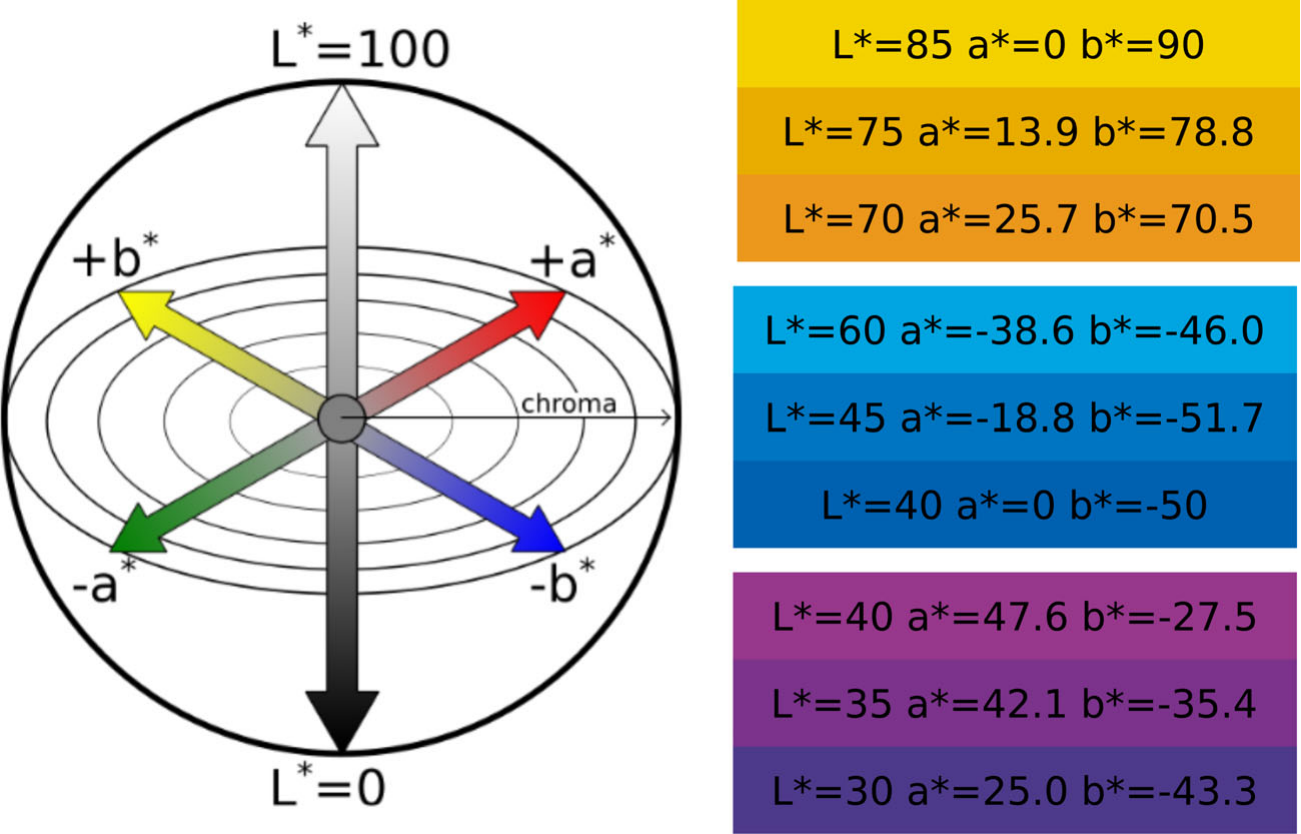
Visual representation of the CIE L*a*b* coordinate with example colours and corresponding CIE values. L* indicates lightness level of the colour, while a* and b* indicate magenta and yellow, respectively, if a* and b* have a positive value and green and blue if the value is negative.

Most plants display a variety of colours and hues in their flowers, ranging from yellow, orange, red and purple to blue, but a few plants show much darker or even black coloration within the petal, such as *Lisianthus nigrescens* (Markham *et al*. 2004). While the occurrence of true black in nature is debated, it was suggest that the definition of black coloration of plants is a function of the CIE (Commission internationale de l'éclairage) L*a*b* coordinate, namely lightness (L*) and chroma [c*: calculated as c* = (a*^2^ + b*^2^)^1/2^] (Van Buren *et al*. 1974; Deguchi *et al*. 2013) (Fig. 2). L* indicates the lightness level of the colour, therefore the lower the L* the darker the colour, while a high L* indicates white. c* is a function of a* and b*, where a* and b* indicate magenta and yellow, respectively, if a* and b* have a positive value, and green and blue if the value is negative. It has been shown, that chroma values are not sufficient to differentiate between purple and black plant cultivars; however, the L* is lower for black plants. This led Deguchi to propose a hypothetical threshold based on L* to determine an objective black definition for plants (Deguchi *et al*. 2013). Previously, Weatherall & Lee (1991) determined the term “black” based on a lightness value below 25 in berries.

**Fig. 3:**
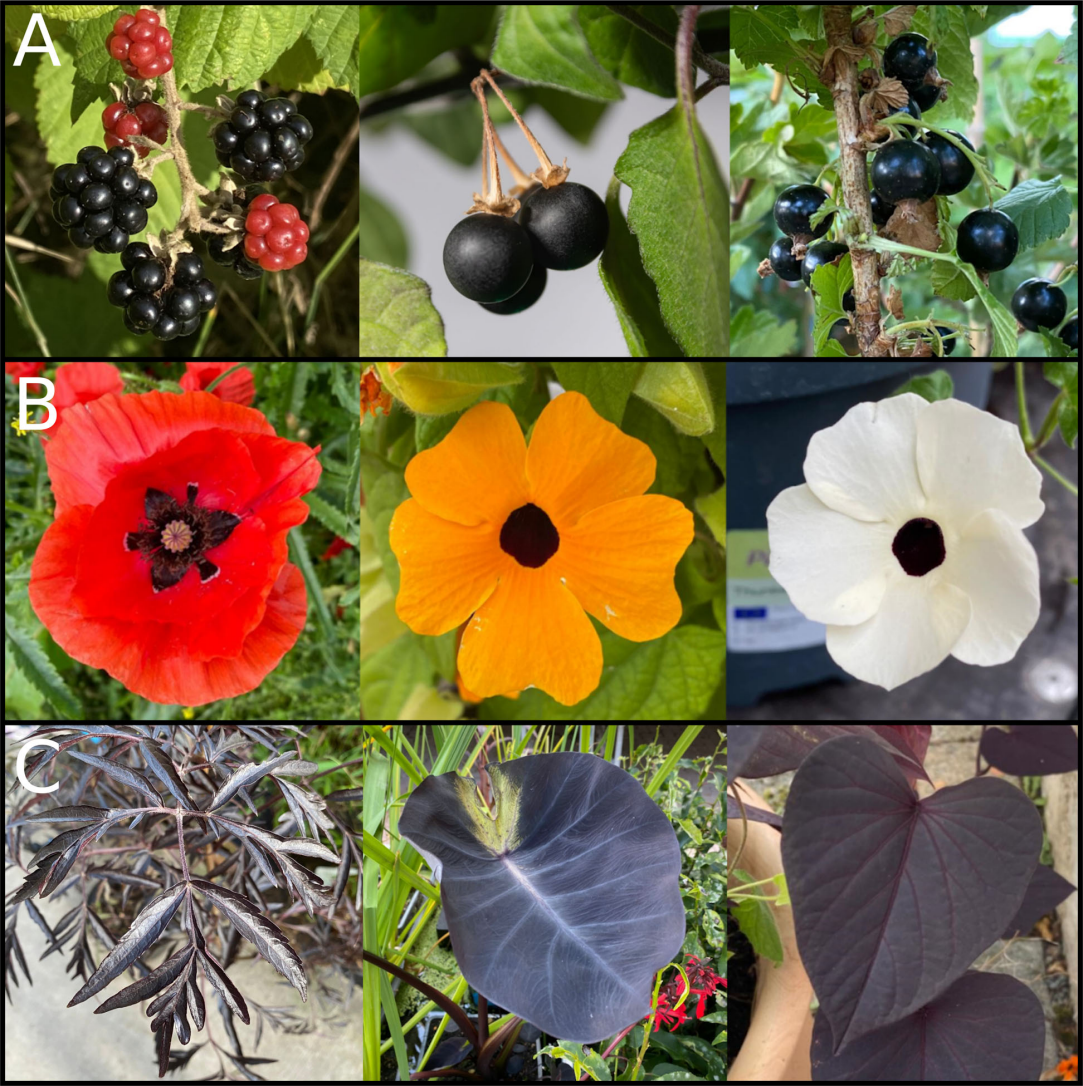
Examples of dark pigmentation in plants. (A) Berries (left to right): blackberry (*Rubus armeniacus*), black nightshade (*Solanum nigrum L*.), black currant (*Ribes nigrum* L.). (B) Petal spots (left to right): poppy (*Papaver rhoeas*), orange black‐eyed Susan vine (*Thunbergia alata*), white black‐eyed Susan vine (*Thunbergia alata*). (C) Leaves (left to right): black elderberry (*Sambucus nigra* ‘black lace’), black elephant ear (*Colocasia rubra* ‘Black Magic’), sweet potato vine (*Ipomoea batatas* ‘SolarTower Black’).

Dark coloration can be found in several plant parts (Fig. 3). For example, the occurrence of black pigmentation in seeds is widespread within the plant kingdom (Glagoleva *et al*. 2020), while dark pigmentation of flowers, fruits, and leaves is rare. The functional implications of this divide are not fully understood and might be associated with varying functions based on the molecular mechanism behind the dark pigmentation. For seeds, there are known mechanisms based on so‐called allomelanins, which is a term describing a group of dark pigments derived from a large number of different potential precursors in plants, such as catechol, caffeic, chlorogenic, protocatechuic, and gallic acids (Glagoleva *et al*. 2020). They often co‐occur with other dark pigments such as oxidized proanthocyanidins, located in the seed coat (Devic *et al*. 1999).

While fully dark pigmentation of flowers, fruits, or leaves is rare, it is not unheard of in nature. Especially within berries there are multiple examples for the occurrence of exceptionally dark fruits. Prominent examples for black fruits are the blackberry (*Rubus armeniacus*), black nightshade (*Solanum nigrum*) (Fig. 3A), mulberry (*Morus nigra*) and black elderberry (*Sambucus nigra*). Additionally, there are several plant species which show partially dark pigmented flowers in the form of seemingly black petal spots and structures. Several potential functions have been proposed for this phenomenon, including an increase in attraction and guidance of pollinating insects, leading to a higher rate of pollination (Sasaki & Takahashi 2002; Davies *et al*. 2012). A well‐known example of petal spots is the poppy (*Papaver rhoeas*), characterized by a bright red petal with black markings at the base of the petal (Fig. 3B) (van der Kooi & Stavenga 2019). Another example for black petal spot formation is *Gorteria diffusa*, which has a bright orange petal colour with a complex dark petal spot towards the base of the petal. The spots increase the flower's attractiveness to potential pollinators (Johnson & Midgley 1997; de Jager *et al*. 2017). Lastly, *Tulipia julia* displays prominent black petal spots at the base of the petals. There are also a few naturally occurring species which display a fully dark flower. One example for a dark to black flower colour would be *Tacca chitari*, commonly known as the bat orchid. This plant belongs to the orchid family and is native within Southeast Asia. Another example is *Lisianthus nigrescens*, a plant native to Mexico and Guatemala, which is regarded as the plant with the darkest petal colour worldwide (Markham *et al*. 2004). In this context, it is noteworthy that there are non‐naturally occurring garden cultivars available which were specifically bred to achieve a black flower colour (Okitsu *et al*. 2018; Hsu *et al*. 2019). There are many plant species available as a dark cultivar, such as tulips, petunias, dahlias, and roses, to name only a few. Furthermore, there are also plant species which naturally display very dark foliage or leaves (Fig. 3C). The dark pigmentation of leaves can be found both in trees as well as grasses. Further, dark pigmentation can also be observed in a variety of grains and cereals (Abdel‐Aal *et al*. 2006). For example, the ancient *Zea mays* subsp. *mays* cultivars “Millo Corvo” and “Nero Spinoso” display a dark phenotype in the kernels (Lago *et al*. 2015; Cassani *et al*. 2017).

**Fig. 4:**
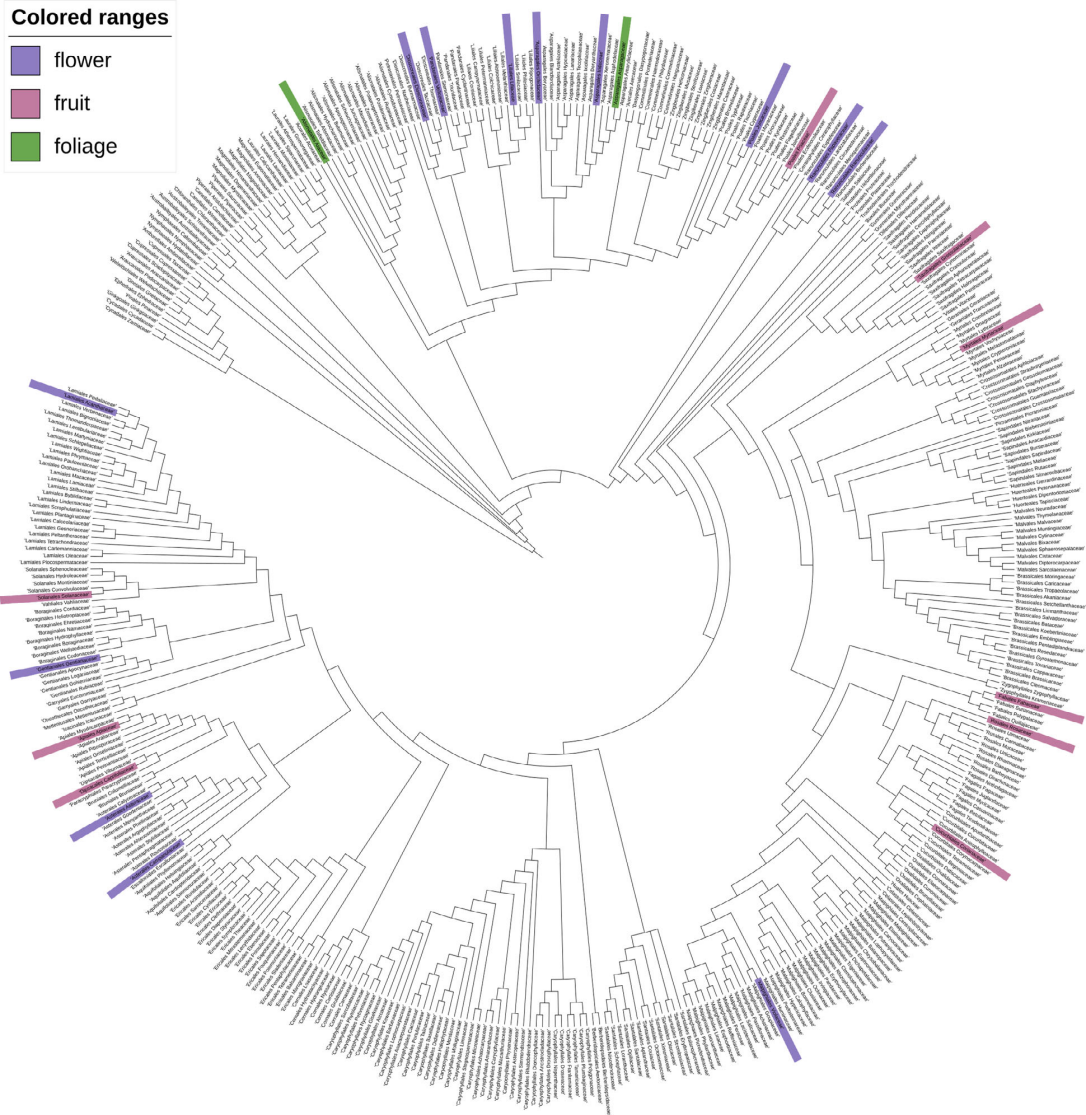
Dark pigmentation occurrence mapped to a phylogenetic tree of plant families. For the highlighted families, at least one species with dark pigmentation has been found. The colours represent the location of the pigmentation: green = foliage, pink = fruit and purple = flower. In total 36 species were included in the highlighting of relevant families. Tree topology is derived from Li *et al*. (2021). A full list of species can be found in File S1.

To understand the phylogenetic distribution of dark pigmentation across plants, an extensive literature screening was performed. The results are summarized and mapped on a phylogenetic family tree (Fig. 4). Families that include at least one species displaying dark pigmentation in any of the selected plant tissue are highlighted. Species which display dark pigmentation can be found in several families. There is no clear clustering of the trait, indicating that it has evolved independently within multiple clades, thus the trait of dark pigmentation appears to be polyphyletic.

### What causes dark pigmentation?

The molecular basis of black pigmentation has been studied for many different species and could be caused by different metabolites in these species. However, many reports revealed a contribution of anthocyanins to the dark pigmentation (Table 2). For example, *Ophiopogon planiscapus* displays black foliage and it has been shown that the chlorophyll content and the anthocyanin content of black leaves grown under sunlight exposure was substantially higher than that of green leaves, while the carotenoid content was lower in black leaves (Hatier *et al*. 2013). The authors present anthocyanins as the most likely explanation for the black leaf colour. Similarly, for many dark‐pigmented plants analyses revealed an exceptionally high content of anthocyanins, ranging up to 24% of petal dry weight for *Lisianthus nigrescens* (Markham *et al*. 2004), leading to the assumption that black colour is induced by the sheer density of anthocyanin accumulation in the petals. However, a study in *Dahlia variabilis* revealed that a sole upregulation of anthocyanin content is not sufficient to induce black colour in petals (Thill *et al*. 2012). It was found that for *D. variabilis*, specific cyanidin derivatives must coincide with a transcriptional block of flavone biosynthesis, thus preventing the formation of flavones to cause black flower colour (Deguchi *et al*. 2013). The presence of anthocyanins alone or a lack of flavone biosynthesis alone were not sufficient to resemble the black flower phenotype. Deguchi *et al*. (2013) speculated that substrate competition between flavone and anthocyanin biosynthesis must be switched off to enable formation of the black phenotype in dahlia. Similarly, studies focused on increasing production of anthocyanins for the establishment of scalable bio‐factories based on plant cell cultivation have shown strong accumulation of anthocyanins within the cells, driven by the introduction of an AmRosea1/AmDelila1 transcription factor complex (Appelhagen *et al*. 2018). Another study revealed that Cy3M5G is more important for black colour than Pg3M5G (Deguchi *et al*. 2016). However, there does not appear to be a consensus as to which anthocyanins are responsible for the black pigmentation, as the main anthocyanin determined for black cultivars varies depending on species (Table 2). For example, a study of the “Sun Black” Tomato line identified petanin, a petunidin‐based anthocyanin, and negretein, a delphinidin‐based anthocyanin, as the most abundant anthocyanins, with petanin making up 56.6% and negretein 21.4% of total anthocyanin content in the black skin of this tomato variety, while no significant amounts of “cyanidin‐based anthocyanins” were detected (Blando *et al*. 2019), directly contradicting the hypothesis that cyanidin derivatives have the strongest impact on black coloration. In summary, these findings indicate that the molecular mechanisms underlying the development of a dark or black phenotype might be species‐specific or at least differ between larger taxonomic groups. This aligns with our phylogenetic analysis of dark to black pigmentation that revealed a polyphyletic nature of this trait (Fig. 4).

**Table 2:** Examples of anthocyanins associated with black pigmentation in fruits and plants.

species	anthocyanin	reference
*Viola tricolor* (pansy)	Delphinidin‐5‐O‐glucoside‐3‐O‐[4‐*p*‐coumaroylrhamnosyl(1–6)‐glucoside]	Goto *et al*. (1978)
*Lisianthus nigrescens* (Flower of Death)	Delphinidin‐3‐O‐rhamnol(1‐6)galactoside	Markham *et al*. (2004)
*Dahlia variabilis* (garden dahlia)	Cyanidin‐3‐(6″‐malonylglucoside)‐5‐glucoside	Deguchi *et al*. (2016)
*Capsicum annuum* (pepper)	Delphinidin‐3‐p‐coumaroyl‐rutinoside‐5‐glucoside	Lightbourn *et al*. (2008)
*Solanum melongena* (Eggplant)	Delphinidin‐3‐(p‐coumaroylrutinoside)‐5‐glucoside (nasunin)	Noda *et al*. (2000)
*Solanum lycopersicum* (Tomato)	Petunidin‐3‐*O*‐[6″‐*O*‐(4^‴^‐*O*‐*E*‐*p‐*coumaroyl‐α‐rhamnopyranosyl)‐β‐glucopyranoside]‐5‐*O*‐β‐glucopyranoside (petanin) Malvidin‐3‐*O*‐[6″‐*O*‐(4^‴^‐*O*‐*E*‐*p‐*coumaroyl‐α‐rhamnopyranosyl)‐β‐glucopyranoside]‐5‐*O*‐β‐glucopyranoside (negretein)	Blando *et al*. (2019)
*Black sorghum* (Sorghum)	Luteolinidin and apigeninidin^a^	Awika *et al*. (2005)
*Phaseolus vulgaris* (Black Bean)	Delphinidin 3‐glucoside, petunidin 3‐ glucoside and maldvidin 3‐glucoside	Wang *et al*. (2013)
*Sambucus nigra* (Black elderberry)	Cyanidin‐3‐O‐sambubioside, prenylated and other derivatives of cyanidin‐3‐acetylrutinoside	Maleknia & Downard (2016)

a Sugar moiety decoration unclear.

### How is the formation of dark pigmentation regulated?

The regulation of anthocyanin metabolism is a complex web involving many different transcription factors and regulators, with the MYB‐bHLH‐WD40 complex being the best studied component (Gonzalez *et al*. 2008, 2016; Albert *et al*. 2023). Understanding the underlying mechanisms can unlock the ability to establish new traits in various plant parts. The following section highlights different regulations associated with the introduction, change, or enhancement of anthocyanin accumulation.

### The role of transcription factors in dark pigmentation

Anthocyanin biosynthesis is regulated by a transcription factor complex composed of three key regulatory proteins: MYB, bHLH, and WD40 (Ramsay & Glover 2005; Gonzalez *et al*. 2008). This complex is referred to as the MBW complex. MYB proteins form the largest family of transcription regulators within plants and are characterized as a highly conserved structure including two or three imperfect repeat motifs of the MYB DNA‐binding motif at the N‐terminus (Dubos *et al*. 2010). Depending on the number of repeats, they are classified, e.g. as R2R3‐MYBs or R3‐MYBs. Each repeat is made up of about 52 amino acids which form three alpha helices each (Rosinski & Atchley 1998). These helices and repeats have regularly spaced tryptophan residues. The third helix is regarded as the “recognition” helix and allows interaction and binding to specific DNA structure (Jia *et al*. 2004). Additionally, MYBs can contain an activation or repression domain at the C‐terminal end, conveying their regulatory function (Dubos *et al*. 2010).

The bHLH transcription factor family is characterized by the structural feature of a basic helix–loop–helix (bHLH) domain and represents the second largest transcription factor family within plants (Murre *et al*. 1994). A stretch of basic amino acids located at the N‐terminal end of the bHLH domain is responsible for the DNA binding, while the helix–loop–helix facilitates protein–protein interaction based on hydrophobic residues (Murre *et al*. 1994). This also enables bHLH transcription factors to be involved in the formation of transcription factor complexes, such as the MBW complex (Lloyd *et al*. 2017).

The WDR family is based on the presence of a WD repeat motif, which is defined as an approximately 40 amino acid structural repeat ending with W‐D (Smith *et al*. 1999). This loose definition makes it much harder to identify WDR proteins, as they can vary greatly in not only the position but also the length of the WDR element. The main function of the WDR proteins is the facilitation of protein–protein interactions to form complexes (Lloyd *et al*. 2017). In the MBW complex, the WDR protein is considered to serve as scaffolding protein for the interaction of a bHLH and a MYB. It has been shown in several species that the MBW is responsible for the activation of the structural genes involved in proanthocyanidin and anthocyanin biosynthesis (Baudry *et al*. 2004; Gonzalez *et al*. 2008). The specificity of the activation is based on the identity of the MYB and bHLH involved in the complex formation.

Several studies aimed to identify the role and identity of transcription factors involved in the occurrence of dark anthocyanin pigmentation. One striking example is the complex formation of petal spots in different cultivars of the Beetle Daisy (*Gorteria diffusa*). By comparing the different cultivars and the metabolites present in the dark petal spots versus the brightly coloured petal, it was revealed that malonylated cyanidins were almost exclusively present in the dark spots as the most abundant anthocyanin (Fattorini *et al*. 2024). Further, three subgroup 6 R2R3‐MYBs, namely *GdMYBSG6‐*1, *GdMYBSG6‐*2, and *GdMYBSG6‐*3, were identified in connection to the spot formation (Fattorini *et al*. 2024). The expression of these transcription factors was significantly increased in spotted tissue and it is assumed that they could act redundantly to promote anthocyanin production, with GdMYBSG6‐2 being the most important (Fattorini *et al*. 2024). Further, it was shown that the expression of *GdMYBSG6‐*1,2,3 leads to upregulation of *GdANS*, *GdDFR*, and *GdMAT1* in spotted tissue compared to unspotted petals (Fattorini *et al*. 2024).

Similarly, in dark *Phalaenopsis* orchids *PeMYB11* was identified as the most likely cause of dark pigmentation (Hsu *et al*. 2019). Here, it was discovered that the dark phenotype was based on the insertion of a retrotransposon, named Harlequin Orchid RetroTransposon 1 (*HORT1*), which further increased expression of *PeMYB11* leading to enhanced activation of the anthocyanin biosynthesis genes (Hsu *et al*. 2019).

## *SOLANUM LYCOPERSICUM*: A MODEL TO UNDERSTAND REGULATION OF ANTHOCYANIN ACCUMULATION IN DARK PHENOTYPES

While the typical tomato plant (*Solanum lycopersicum*) does not produce anthocyanins in fruit, wild relatives, such as the wild *Solanum* species *S. chilense* and *S. lycopersicoides*, are capable of producing and accumulating anthocyanins in the fruit skin. *Solanum chilense* develops a distinct phenotype of dark‐spotted fruits on green fruit skin in response to low temperatures or high light exposure (Mes *et al*. 2008). The genetic region responsible for the induction of anthocyanin synthesis is the *Anthocyanin Fruit* (*Aft*) locus, located on chromosome 10 and encoding a R2R3‐MYB (Colanero *et al*. 2020). The MYB has been identified as SlAN2‐like and has been shown to activate expression of *DFR* based on the formation of a MBW complex consisting of SlAN2‐like, WDR, bHLH1, and bHLH2 (Sun *et al*. 2020). Comparison of the SlAN2‐like sequence with that of a red‐fruited tomato revealed a small sequence variation leading to alternative splice sites (Sapir *et al*. 2008). While the spliced version from *S. chilense* leads to a functional R2R3‐MYB, the alternatively spliced SlAN2‐like in *S. lycopersicum* cannot activate the anthocyanin biosynthesis pathway (Colanero *et al*. 2020). If SIAN2‐like from *S. chilense* is introduced into *S. lycopersicum* an accumulation of anthocyanins in the fruit skin can be observed, further indicating SlAN2‐like is a key factor for the introduction of anthocyanin biosynthesis in *Solanum* plants (Sapir *et al*. 2008). A similar mechanism was discovered for *Solanum lycopersicoides*, with the responsible locus called ‘*Aubergine*’ or *Abg* (Rick *et al*. 1994; Mes *et al*. 2008). Homozygous plants for *abg* develop infertile, deformed, very dark fruits, while heterozygotes produce fruits with the expected shape and size but still presenting a dark phenotype. Menconi *et al*. (2023) identified the location of *Abg* on Chromosome 10 and detected the same splice site variation as in *Aft*, along with another splicing variant leading to reduced anthocyanin accumulation. Further, the absence or reduced activity of an anthocyanin repressor, SlMYB‐ATV, may play a role in the accumulation of anthocyanins in *Solanum* species. For the genetically engineered purple tomato species Indigo rose, a 4 bp deletion in the repressor led to a significant increase in anthocyanin accumulation, while other dark tomato varieties with an intact repressor showed a lower anthocyanin content (Cao *et al*. 2017). These studies of different close relatives of tomato demonstrate that the capacity to produce anthocyanins was lost following the introduction of an alternative splicing site in the R2R3‐MYB gene *SlAN2‐like*, an activator of late anthocyanin biosynthesis.

## GENETIC MODIFICATION RESULTING IN A DARK PHENOTYPE

Besides naturally occurring dark phenotypes, there have been attempts to introduce the phenotype via genetic engineering (Zhang *et al*. 2014, 2016). The main interest of the studies was the health‐promoting properties of anthocyanins, with the introduction of a dark phenotype being a secondary effect. There are two distinct approaches and targets for the enrichment of anthocyanins. The first target is to produce dark phenotypes in commonly consumed fruits and vegetables, such as the tomato. As previously described, there have been long‐standing breeding programs with naturally occurring anthocyanin‐producing wild species to generate a dark tomato cultivar (Mazzucato *et al*. 2013). The success through breeding avoids the production of a genetically modified plant, making it particularly interesting for the European market. Simultaneously, the use of genetic engineering has also been applied to induce the production of anthocyanins within the tomato clade (Butelli *et al*. 2008, 2021).

The introduction of anthocyanin biosynthesis is based on the introduction of new regulatory genes encoding transcription factors activating the relevant anthocyanin biosynthesis genes. By introducing the bHLH protein Delila (AmDel) and the MYB protein Rosea1 (AmRos1) to a wild‐type tomato species, anthocyanin biosynthesis is reactivated and the dark phenotype is introduced (Butelli *et al*. 2008). The transgenes are under control of the fruit‐specific E8‐promoter resulting in accumulation within the entire fruit, both pulp and skin, leading to an increased amount of anthocyanins in comparison to the bred purple tomato varieties previously described, which only accumulate anthocyanins within the skin (Butelli *et al*. 2008; Mazzucato *et al*. 2013). Del and Ros1 activate several genes required for anthocyanin biosynthesis, including *PAL*, *CHS*, and *DFR*, explaining the high accumulation of anthocyanins (Butelli *et al*. 2008; Naing *et al*. 2017). This leads to a dark purple phenotype. An additional expression of *MYB12* from *Arabidopsis thaliana* resulted in increased accumulation of anthocyanins, leading to a more intensely coloured tomato fruit, because of enhanced activation of *PAL* and *CHS*; this variant is referred to as ‘indigo’ (Butelli *et al*. 2021). A similar approach has been used to establish microbial cell factories, in order to build stable high‐yield production of specifically decorated anthocyanins for application in the food industry as colourants or food additives. Appelhagen *et al*. (2018) presented a strategy to induce anthocyanins in *Nicotiana tabacum* cv. Samsun cells, by introducing the bHLH Delila (AmDel) and the MYB Rosea1 (AmRos1) from *Antirrhinum majus*. In *N. tabacum* the accumulation of anthocyanins is restricted to flower petals, while producing almost exclusively cyanidin 3‐O‐rutenoside (C3R) (Appelhagen *et al*. 2018). When the *AmRos1* and *AmDel* are constitutively expressed in *N. tabacum* plants, the anthocyanin accumulation expands to the rest of the plant, with C3R remaining the main anthocyanin produced (Appelhagen *et al*. 2018). A cell culture was established and showed stable production throughout the cultivation, reaching anthocyanin levels higher than the amount of C3R found within anthocyanin‐rich fruits, such as blackberries or blueberries (Appelhagen *et al*. 2018). Furthermore, by introducing additional genes like the F3′5′H from *Petunia* or 3‐O‐rutinoside‐4″′‐hydroxycinnamoyl transferase from *Solanum lycopersicum* (Sl3AT), the range of anthocyanins can be expanded, allowing production of several anthocyanins at the same time (Appelhagen *et al*. 2018).

### Stability of anthocyanin pigmentation

The stability of anthocyanins can be influenced by a variety of parameters, such as the pH, temperature, light conditions, co‐pigmentation with flavones and iron, as well as additional modifications, such as glycosyl and aromatic or aliphatic acyl moieties (Rowan *et al*. 2009; Zhao *et al*. 2021). For example, light conditions are an important factor to consider when storing anthocyanins, as light has a significant negative effect on their stability (Kearsley & Rodriguez 1981; Amr & Al‐Tamimi 2007). By storing anthocyanin extracts in the dark, the half‐life of the molecules is significantly increased. A study showed that extracts from blackcurrant fruits lose half of their antioxidant potential and associated anthocyanins within 8.25 days when stored in natural light conditions (Jia *et al*. 2013). Under artificial light, the half‐life is increased to 18.81 days, while storage in complete darkness further increases the half‐life to 21 days (Jia *et al*. 2013). Similar results were reported for extracts from both mulberry and acai berry (de Rosso & Mercadante 2007; Aramwit *et al*. 2010).

Moreover, high temperature has also been reported to significantly decrease the stability of anthocyanins, with the anthocyanin content of grape extracts being reduced to less than half (Mori *et al*. 2007). For blueberries it has been reported that the half‐life in pH 3.0 of anthocyanins slowly decreased with a temperature increase from 25°C to 50°C (Liu *et al*. 2018). However, further increasing the temperature to 60°C and above leads to a rapid decrease in half‐life, resulting in ¼ of the half‐life at 60°C compared to 25°C (Liu *et al*. 2018).

Interestingly, not all anthocyanins appear to degrade at the same rate. In a study focusing on the stability of anthocyanins in black carrot, it was found that acylated anthocyanins from black carrot remain more stable to temperature increase of 20–50°C compared to non‐acetylated anthocyanins extracted from blackberry (Zozio *et al*. 2011). Similarly, diacylated anthocyanins provide significantly high blue colour stability to red cabbage at 50°C as compared to non‐acylated anthocyanins (Fenger *et al*. 2021). However, the increased stabilization of anthocyanins through acylation is dependent on the type of acyl group, their position or attachment side, as well as the number of acylations (Fenger *et al*. 2021). In general, it is differentiated between aromatic and aliphatic acyl groups. The stabilization based on acylation by aromatic acid is based on enabling intra‐ and intermolecular co‐pigmentation or self‐association of anthocyanins. However, the stabilization of anthocyanins by acylation with aliphatic acid is based on the introduction of steric hindrance (Jokioja *et al*. 2021).

However, the effect of the stabilization is highly dependent on the identity of the acylation added. Luo *et al*. (2007) showed that, for anthocyanins extracted from a transgenic tobacco line, the highest stability of anthocyanin was conveyed by the addition of (coumaroyl) glucoside with a half‐life of 48 h, followed by cyanidin 3‐rutinoside (*t*½ = 24 h), cyanidin 3‐glucoside (*t*½ = 10 h) and, lastly, cyanidin 3‐glucoside 5‐malonylglucoside (*t*½ = 5.5 h) (Luo *et al*. 2007).

### What are the proposed ecological functions and potential industrial applications of dark pigmentation?

**Fig. 5:**
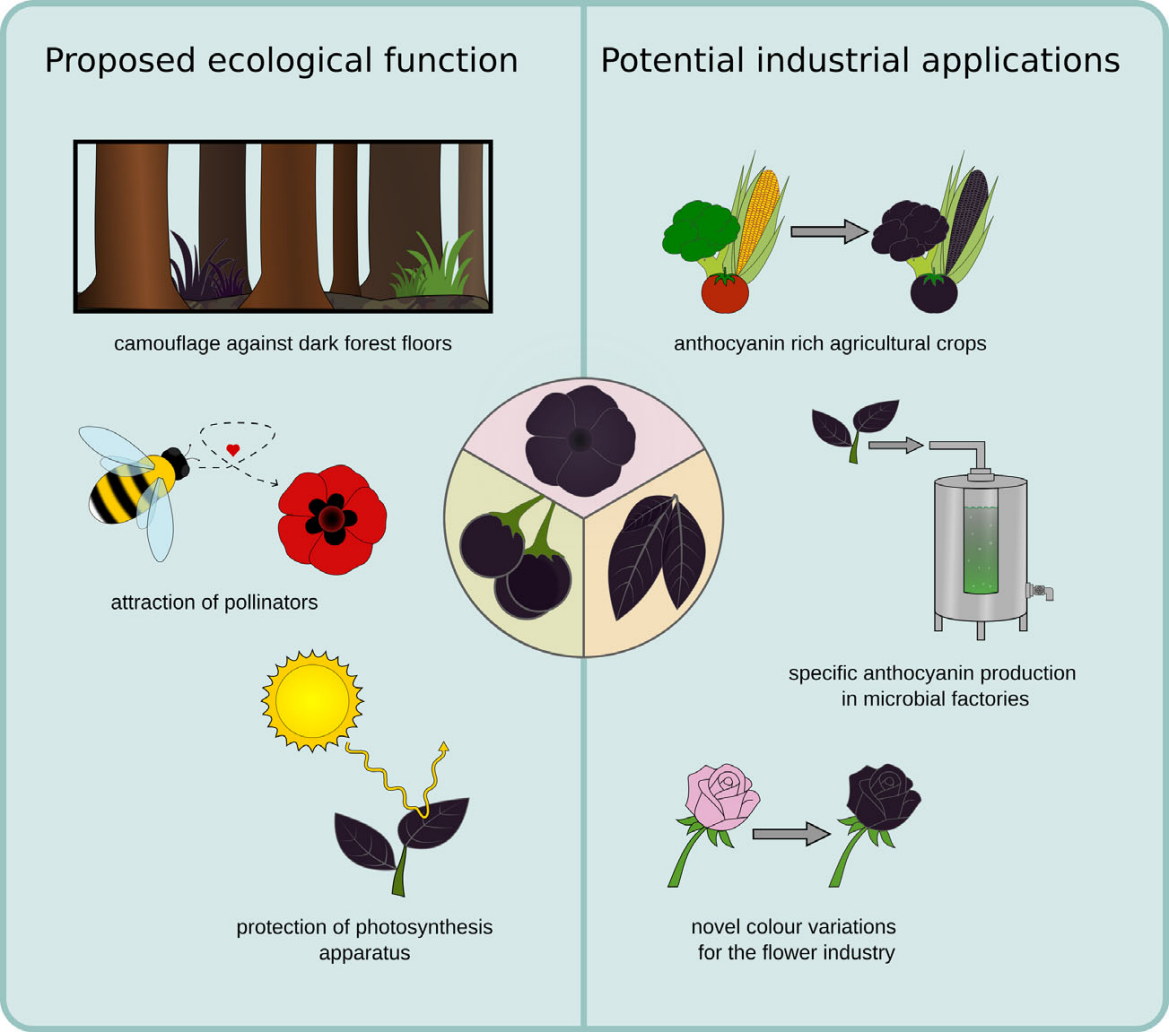
Schematic overview of proposed ecological functions and potential industrial applications of dark pigmentation.

Anthocyanins have been associated with many beneficial characteristics based on their antioxidant properties, and serve several essential functions which contribute to plant survival, development, and interaction with the environment (Chalker‐Scott 1999; Gould 2004). Additionally, vibrant colours give plants an advantage in attracting different pollinators. However, the repeated occurrence of dark petals, foliage, and berries suggests that there are also potential benefits to dark colours. One proposed advantage of black foliage is indirect protection against herbivores, as the dark foliage looks reminiscent of a dead plant and is, in general, less visible to herbivores against exposed soil of a dark forest floor (Fig. 5) (Gould 2004). This could potentially explain the occurrence of dark leaves and foliage as seen in *Ophiopogon planiscapus*. Furthermore, it has been proposed that dark flowers might help with pollination due to an increased nectar temperature caused by the poor reflection of sunlight which warms the flower (Büdel 1959; Tikhomirov *et al*. 1960; Lacey *et al*. 2010). This could be beneficial in cold environments, as insects are more likely to visit dark flowers in these conditions (Norgate *et al*. 2010). A study involving the Australian native bee confirmed that an increased nectar temperature is only an advantage if the environmental temperature is low (Norgate *et al*. 2010). However, a test on *O. planiscapus* did not show a significant influence of the dark pigmentation on the leaf temperature, while simultaneously revealing that intense pigmentation of the leaves reduces carbon fixation and biomass formation under intense light (Hatier *et al*. 2013). At the same time, the photoinhibitory stress was lowered (Gould, Neill, *et al*. 2002; Hatier *et al*. 2013). These findings suggest that dark pigmentation has potential to reduce photosynthesis of a plant while simultaneously providing protection of the photosynthetic apparatus. While the mechanism for the enhanced photoprotection in the black leaves of *O. planiscapus* is not clear (Hatier *et al*. 2013), it is most likely related to high accumulation of anthocyanins, as the functions of anthocyanins are protection against UV radiation from the sun and scavenging reactive oxygen species (ROS) (Gould 2004). Anthocyanins act as a natural sunblock, by absorbing UV‐B and UV‐A light (Landi *et al*. 2015). This protects plant tissue from potential negative effects of UV rays and is particularly important for plants in areas with intense sunlight and at high altitudes (Gould 2004). Further, they could provide protection through the absorbance of excess photons while additionally being antioxidants that scavenge (ROS and prevent damage to the photosynthesis apparatus; Fig. 5) (Gould 2004).

Another natural function of anthocyanins lies in the attraction of pollinators and seed dispersers. For example, several plant species utilize dark pigmentation in order to mimic their respective pollinator in order to attract them to the flower (Fig. 5) (Johnson & Midgley 1997). Examples for this mimicry or sexual deception can be observed for the beetle daisy as well as the genus *Ophrys*. The dark petal spot of *G. diffusia* mimics the bee fly *Megapalpus capensis* as this is their main pollinator, with male flies preferring the more complex petal spots (Thomas *et al*. 2009; Ellis *et al*. 2014). Similarly, the genus *Ophrys* imitates several different species of insect pollinators and several shifts and adaptations to new pollinators can be traced within this genus (Breitkopf *et al*. 2015). The *Ophrys* genus displays an exceptionally high rate of speciation, which has led to the emergence of several hundred species in the Mediterranean region of the western Palaearctic (Baguette *et al*. 2020). By imitating sexually receptive females of one particular species of insect, the *Ophrys* genus is capable of attracting male pollinators and thus increasing their pollination rate. Both the structure of the flower as well as the dark pigmentation play key roles in this process (Bradshaw *et al*. 2010). Additionally, in berries, the anthocyanin content often increases with ripening of the berry. For example, blackberries change from light green to black over the course of ripening. Although red pigmentation often appears more effective, the darker colour attracts birds, who consume the berries and then distribute the seeds (Schaefer *et al*. 2006; Duan *et al*. 2014; Enaru *et al*. 2021).

Anthocyanin production greatly increases in the presence of stress factors (Chalker‐Scott 1999; Liu *et al*. 2020), particularly in response to low temperatures (Christie *et al*. 1994), drought (Chalker‐Scott 1999), nutrient deficiencies (Peng *et al*. 2008), or pathogen attacks (Liu *et al*. 2020). Their connection to stress response is assumed to be due to their antioxidant properties (Gould, McKelvie, *et al*. 2002). These antioxidant properties are what makes anthocyanins interesting to the field of medicine, and anthocyanins have been implicated as preventive or curative measures for several health conditions, such as cardiovascular disease, diabetes, and obesity (Belwal *et al*. 2017; Lee *et al*. 2017; Dong *et al*. 2022).

While certain pigmented grain and cereal varieties, such as black rice cultivars, contain high levels of anthocyanins, and their extracts have been shown to have antioxidant activity, the full potential of these effects cannot be conveyed with regular consumption (Bordiga *et al*. 2014; Lago *et al*. 2015; Mbanjo *et al*. 2020). Similarly, anthocyanins are often present in commonly consumed fruits and vegetables, their concentration and therefore uptake may not be sufficient to provide the abovementioned health benefits (Butelli *et al*. 2008).

In order to overcome this challenge, several attempts have been made to increase the anthocyanin concentration in popular agricultural crops (Butelli *et al*. 2008; Petroni *et al*. 2014). While there are approaches to selectively breed for the desired increase in anthocyanin concentration, this is very time consuming (Mazzucato *et al*. 2013). Alternatively, for the tomato plant, the increase in anthocyanins and induction of a dark phenotype was achieved by introduction of novel transcription factors through genetic engineering, thus allowing more targeted introduction of desired anthocyanins (Butelli *et al*. 2021). Another interesting industrial application for determining the mechanism of dark pigmentation is that it would allow targeted introduction of a dark phenotype into ornamental plants or flowers for the cut flower industry. Previously, there have been studies introducing novel colour variants of *Chrysanthemum morifolium* Ramat. By expanding the anthocyanin biosynthesis pathway to include delphinidin‐derived anthocyanins, a blue and purple phenotype was introduced into this species (Noda *et al*. 2013). A similar approach can be followed to expand colour variations within desirable ornamental plants to include a dark phenotype.

The natural colour properties of anthocyanins make them highly desirable as alternatives to synthetic dyes in various industries, including food, cosmetics, textiles, and nutraceuticals (Alappat & Alappat 2020). In the food industry, anthocyanins are used as natural colourants to enhance the visual appeal of a wide range of products, such as beverages, dairy products, confectionery, and baked goods (Appelhagen *et al*. 2018; Ghosh *et al*. 2022). However, despite their promising scientific and industrial potential, there are several challenges associated with the industrial use of anthocyanins. For example, the currently commercially available colour spectrum of anthocyanins is still limited (Appelhagen *et al*. 2018). At the moment, anthocyanins are extracted from waste products from the wine industry, such as grapes or vegetables such as red cabbage and purple sweet potato (Francis & Markakis 1989; Li *et al*. 2019; Ghareaghajlou *et al*. 2021). While this presents an economically useful process, it also means that no batch of extracted anthocyanins will be exactly the same. Additionally, it is not possible to easily extract just one specific anthocyanin with the desired colour properties, but rather a mix of all anthocyanins present in the fruit in varying quantities. This leads to slight colour variation between batches of extracted anthocyanins (Díaz‐García *et al*. 2015).

Anthocyanins also provide an attractive alternative to synthetic dyes, as they are perceived as natural and healthier options by consumers who prefer “clean‐label” products. In regard to dark food coloration, a clean alternative to synthetic colourants is currently lacking. To the best of our knowledge, the only available natural black food colourant on the market is vegetable carbon or E153. This food colourant is created by charring wood and vegetable parts to turn them into activated charcoal, which is added to foods to convey black coloration (EFSA Panel on Food Additives and Nutrient Sources added to Food (ANS) 2012). The use of vegetable carbon, as any food additives, is under strict regulation by several authorities, such as the FDA in the United States or the EFSA in Europe. However, there are consumer concerns about the potential presence of carcinogenic compounds within E153 (Hilber *et al*. 2022). Answering the scientific question regarding the underlying mechanism of dark pigmentation in plants could enable the production of a natural colourant allowing substitution of E153. In addition to the food industry, anthocyanins are similarly used in the cosmetics and textile industry to produce natural colourants which meet the current consumer demand for sustainable and non‐toxic alternatives (Gebhardt *et al*. 2020).

## CONCLUSION AND FUTURE PERSPECTIVES

Dark pigmentation in plants is a rare and fascinating phenomenon, with many unanswered research questions. Currently, strong expression and accumulation of anthocyanins is the most likely explanation for its occurrence. However, the reason behind this accumulation, the underlying mechanism, and the identity of the necessary anthocyanin are not yet fully resolved.

The main anthocyanin for dark phenotypes varies from species to species, indicating that there are different mechanisms leading to dark colour. This is also supported by the independent evolution of dark phenotypes across different plant families.

Additionally, several mechanisms for regulation associated with overexpression of anthocyanins have been identified. One mechanism is based on repression or knockout of the competing flavone biosynthesis, leading to darker pigmentation, while another mechanism is based on activation and upregulation of the anthocyanin biosynthesis genes in the presence of MYB transcription factors. However, there are still several key questions which should be addressed to fully understand the occurrence of dark pigmentation in plants. First, the composition of the anthocyanins present within dark‐pigmented plant parts should be analysed and compared between closely related species. Is there a consensus on the base anthocyanin or the modifications present? Further, it is necessary to shed light on both the genes involved in the production of the anthocyanins associated with dark pigmentation and also the regulatory elements involved. Understanding the underlying cause of dark pigmentation in plants will impact several industrial applications.

By revealing the regulatory elements and genes involved in this distinct phenotype it opens up the possibility of either selectively breeding or screening for the desired traits in flowers or even inducing these traits with the help of genetic engineering or genome editing. Being able to develop dark flowers for popular ornamental plants would be an interesting application for the cut flower industry. Similarly, this approach could be expanded to agriculturally interesting plants, as shown in tomato, to increase the anthocyanin content and therefore the antioxidant properties of the targeted crop. This could lead to health benefits for consumers or be utilized to increase the yield of anthocyanin extractions in foods for application in natural food colourants. Additionally, this would unlock heterologous production of specific anthocyanins within yeast or the establishment of a plant cell culture. Heterologous expression is a promising approach for targeted production of specific anthocyanins associated with dark coloration, with the potential of establishing upscaling to industrial standards and parallelization, leading to increased yield of natural colourants for use in the food industry.

## AUTHOR CONTRIBUTIONS

BP and KW conceived the work. KW conducted analyses and created the figures. BP and KW wrote the manuscript. All authors read and approved the manuscript.

## Supporting information


**File S1.** List of plant species exhibiting dark pigmentation. highlighted in the phylogenetic tree (Fig. 4). This figure presents all plant species identified as displaying dark pigmentation and highlighted in the phylogenetic tree in Fig. 4. For each species, the taxonomic classification is provided, including order, family, genus, and species. Additionally, the specific tissue exhibiting dark coloration is indicated ‐ flower, fruit, or foliage.
